# Yogurt Fortification by the Addition of Microencapsulated Stripped Weakfish (*Cynoscion guatucupa*) Protein Hydrolysate

**DOI:** 10.3390/antiox10101567

**Published:** 2021-10-01

**Authors:** Karina Oliveira Lima, Meritaine da Rocha, Ailén Alemán, María Elvira López-Caballero, Clara A. Tovar, María Carmen Gómez-Guillén, Pilar Montero, Carlos Prentice

**Affiliations:** 1 Laboratory of Food Technology, School of Chemistry and Food, Federal University of Rio Grande (FURG), Rio Grande 96203-900, RS, Brazil; karinah_ol@hotmail.com (K.O.L.); dqmprent@furg.br (C.P.); 2 Laboratory of Microbiology, School of Chemistry and Food, Federal University of Rio Grande (FURG), Santo Antônio da Patrulha 95500-000, RS, Brazil; meritaine@gmail.com; 3 Institute of Food Science, Technology and Nutrition (ICTAN-CSIC), 28040 Madrid, Spain; ailen@ictan.csic.es (A.A.); cgomez@ictan.csic.es (M.C.G.-G.); 4 Department of Applied Physics, University of Vigo, As Lagoas, 32004 Ourense, Spain; tovar@uvigo.es

**Keywords:** fish protein hydrolysate, microencapsulation, yogurt, physicochemical properties, antioxidant activity, antihypertensive activity

## Abstract

The aim of the present work was to fortify yogurt by adding a stripped weakfish (*Cynoscion guatucupa*) protein hydrolysate obtained with the enzyme Protamex and microencapsulated by spray drying, using maltodextrin (MD) as wall material. The effects on the physicochemical properties, syneresis, texture, viscoelasticity, antioxidant and ACE inhibitory activities of yogurt after 1 and 7 days of storage were evaluated. In addition, microbiological and sensory analyses were performed. Four yogurt formulations were prepared: control yogurt (without additives, YC), yogurt with MD (2.1%, YMD), with the free hydrolysate (1.4%, YH) and the microencapsulated hydrolysate (3.5%, YHEn). Yogurts to which free and microencapsulated hydrolysates were added presented similar characteristics, such as a slight reduction in pH and increased acidity, with a greater tendency to present a yellow color compared with the control yogurt. Moreover, they showed less syneresis, the lowest value being that of YHEn, which also showed a slight increase in cohesiveness and greater rheological stability after one week of storage. All yogurts showed high counts of the microorganisms used as starters. The hydrolysate presence in both forms resulted in yogurts with antioxidant activity and potent ACE-inhibitory activity, which were maintained after 7 days of storage. The incorporation of the hydrolysate in the microencapsulated form presented greater advantages than the direct incorporation, since encapsulation masked the fishy flavor of the hydrolysate, resulting in stable and sensorily acceptable yogurts with antioxidant and ACE inhibitory activities.

## 1. Introduction

Milk and dairy products, such as yogurt, are widely appreciated and consumed worldwide because of their sensory and nutritional characteristics [1,2]. Compared with milk, yogurt is more nutritious and an excellent source of protein, calcium, phosphorus, riboflavin, thiamine, vitamin B12, niacin, magnesium, and zinc [3]. However, despite their beneficial health effects, these products are generally not considered to be an important source of bioactive compounds [2].

There are some studies related to the addition to yogurt of bioactive compounds, such as *Spirulina platensis* [4,5], rice bran [6], strawberry pulp [1], mushroom extracts (*Agaricus bisporus*) [7], fish collagen [8], monk fruit extract (*Siraitia grosvenorii*) [9] and *Ficus glomerata* Roxb fruit extract [10], in which the addition of these compounds increased the antioxidant and/or angiotensin-converting enzyme (ACE) inhibitory capacity. This may be an ideal strategy for facilitating the consumption of bioactive compounds and increasing the functionality of food, as people of all ages widely accept and consume yogurt.

Correspondingly, proteins derived from fish by-products represent a very interesting source of bioactive peptides due to their low cost and the established requirement of reducing agro-industrial waste [11]. Fish protein hydrolysates are sources of peptides with diverse bioactivities, including antioxidant and antihypertensive actions [12]. Some peptides present antihypertensive activity as they are able to inhibit the angiotensin-converting enzyme (ACE), which has a fundamental role in the regulation of blood pressure [12,13,14]. Moreover, antioxidants can also delay oxidative stress, involved in the occurrence of various diseases, including hypertension and aging [15].

To improve bioactivity, protein hydrolysates and bioactive peptides can be incorporated into different foods, such as dairy products, for their functional properties. However, scarce information about the incorporation of fish hydrolysates into yogurt is available. In one study, bovine and fish skin hydrolysates induced greater syneresis and less firmness and viscoelasticity in skimmed bovine milk yogurt than caseinate hydrolysate, which could be attributed to their hindering effects on yogurt acidification [16]. Moreover, the incorporation of fish protein hydrolysates can be difficult due to their high hygroscopicity, bitter taste, chemical instability, interaction with the food matrix, incompatibility and limited bioavailability [17,18,19]. Microencapsulation is a process in which the compounds of interest are coated or incorporated into a protective matrix, and is considered effective for overcoming the limitations mentioned above [20].

Among the numerous encapsulation techniques, spray drying is widely used in the food industry [21,22]. This technique consists of atomizing a formulation containing the protective matrix and bioactive compounds in small drops, followed by subsequent rapid drying through a stream of hot air to produce dry microparticles [14,23]. In previous studies, Lima et al. [24] used spray drying to encapsulate a stripped weakfish hydrolysate in maltodextrin. The microencapsulated hydrolysate was characterized, and the stability and biological properties were evaluated in vitro and in vivo in a model of *Caenorhabditis elegans*; the results showed improvements in growth and reproduction rate as well as a protective effect on nematodes exposed to oxidative stress upon consumption of the encapsulates.

Based on the above-mentioned information, the objective of this study was to develop a functional yogurt by incorporating a microencapsulated protein hydrolysate from stripped weakfish (*Cynoscion guatucupa*) in the formulation. For this purpose, sensory and microbiological analyses were performed to confirm that a quality product was obtained. Subsequently, the effects on the physicochemical and rheological properties, texture, and bioactivity of the yogurts after 1 and 7 days of storage were evaluated.

## 2. Materials and Methods

### 2.1. Materials

The by-products of stripped weakfish (*Cynoscion guatucupa*) were obtained from a fishing company in the city of Rio Grande (RS, Brazil). The carcasses and trimmings were processed in a meat–bone separator (High Tech, HT250C, Chapecó, Brazil), discarding the skin and bones. The resulting muscle was packed in plastic bags and stored at −18 °C until use. Protamex enzyme purchased from Sigma-Aldrich (St. Louis, MO, USA) was used in the hydrolysis process. Maltodextrin with a dextrose equivalent (DE) of 5 was purchased from Manuel Riesgo S.A. (Madrid, Spain). All chemical reagents used in this study were of analytical grade.

### 2.2. Protein Hydrolysate

The fish muscle protein hydrolysate, with a degree of hydrolysis of 5%, was produced using the enzyme Protamex at 50 °C and pH 7 as previously described in Lima et al. [24]. The lyophilized hydrolysate was stored at −18 °C until use. Hydrolysate characteristics (amino acid profile, Fourier transform infrared spectroscopy, morphological and biological activity) were previously described in Lima et al. [24,25].

### 2.3. Microencapsulation

The hydrolysate was microencapsulated with maltodextrin (MD) by spray drying as described by Sarabandi et al. [21], with some modifications. The MD and the hydrolysate (60:40 *w*/*w*) were dissolved in distilled water constituting 10% of the total solids and stirred for 3 h at room temperature. Subsequently, the solutions were atomized in a Mini Spray Dryer B-290 (Büchi, Switzerland) at 0.3 L/h with an inlet temperature of 130 °C, aspiration rate 100% and an outlet temperature of 70 ± 2 °C. Thus, a microencapsulated hydrolysate (microcapsules composed of maltodextrin and hydrolysate) was obtained. The characteristics and stability of the encapsulation have been previously described in Lima et al. [24].

### 2.4. Yogurt Preparation

Yogurts were made with UHT (Ultra High Temperature) whole cow milk (fat 3.6%; protein 3.1% and carbohydrates 4.6%) and natural yogurt was purchased from a local market (Madrid, Spain). Yogurts were prepared in a Thermomix (Vorwerk & Co., Wuppertal, Germany). Firstly, the milk was heated to 40–45 °C while stirring for 3 min, followed by the addition of natural yogurt and stirring for 5 min. Later, the tested ingredients were added, and the mixture was subsequently stirred for another 5 min to constitute the different samples. The temperature was maintained during the mixing steps. For each 100 mL of milk, 12.5 g of natural yogurt and different concentrations of the tested ingredients were added, in order to maintain the same concentration of wall material and hydrolysate present in the microencapsulated hydrolysate. Four lots of yogurts were then obtained: control yogurt (without additional ingredients; lot YC); yogurt with the addition of 2.1 g maltodextrin (wall material; lot YMD); yogurt with the addition of 1.4 g of free protein hydrolysate (lot YH) and yogurt with the addition of 3.5 g of microencapsulated hydrolysate (lot YHEn, composed of 2.1g MD and 1.4 g of hydrolysate). Later, the formulations were transferred to disposable plastic cups of 100 mL and incubated at 43 °C until reaching pH 4.6 [26]. The yogurts were stored under refrigeration (6 ± 1 °C) for 7 days.

### 2.5. Yogurt Characterization

#### 2.5.1. Proximal Analyses, Physicochemical Analysis and Color

Proximate composition (moisture, ash, protein, and fat) was evaluated following the official methods AOAC 19,927.05, AOAC 945.46, AOAC 991.22, and AOAC 905.02, respectively [27]. Carbohydrate content was estimated by difference. Protein content was estimated by nitrogen determination using the factor of 6.25. The pH was measured by a digital pH-meter (Methrom 827, Herisau, Switzerland), previously calibrated, at room temperature. The titratable acidity (TA) of the yogurts was determined by titrating 9 g of the sample with 0.1 M NaOH using phenolphthalein as an indicator, which was expressed as % lactic acid. Both determinations were performed in triplicate. The color of the yogurts was measured in a Konica Minolta CM-3500d spectrophotometer (Konica Minolta Sensing, Inc., Osaka, Japan) from ten measurements of each sample, and the CIELAB color space was used to obtain the color coordinates. The color was expressed by the parameters L*, a* and b*. The whiteness index (WI) was determined according to Equation (1):

(1) $$\mathrm{WI}=100-\sqrt{{\left(100-L\right)}^{2}{+a}^{2}{+b}^{2}}$$

#### 2.5.2. Syneresis

The syneresis of the yogurts was determined according to Santillán-Urquiza et al. [28]. Approximately 10 g of yogurt was centrifuged at 176× *g* for 20 min at 10 °C. The syneresis, expressed as percentage, was performed in triplicate and estimated as the weight of the supernatant released over the weight of the initial yogurt × 100.

#### 2.5.3. Texture Analysis

Firmness and cohesiveness parameters were determined using a texture analyzer (TA.XTplus, Stable Micro Systems) as described by Santillán-Urquiza et al. [28], with modifications. Briefly, the compression force (N) in 50 mL of yogurt was measured using a 36 mm diameter × 50 mm high cylindrical body (P36R), with a speed of 0.5 mm/s and reaching a depth of 20 mm. Three different yogurt cups were measured for each lot and the firmness results were expressed in N.

#### 2.5.4. Rheological Analyses

Oscillatory shear measurements were performed on a Bohlin CVO rheometer (Bohlin Instruments Ltd., Gloucestershire, UK) using a cone-plate geometry (4° angle, 40 mm diameter, 0.15 mm gap). The temperature in the lower plate was 5 °C. Frequency sweep tests were carried out over a range of angular frequencies between 0.63 and 63 rad/s with an oscillation strain of 5%, selected from the linear viscoelastic region (LVER). The storage modulus (*G*′) and loss modulus (*G*″) were plotted as a function of angular frequency (ω).

To understand the viscoelastic properties of the different yogurts, flow and viscoelastic properties of the additive aqueous suspensions were examined. Thus, three aqueous suspensions at the same concentration (1.5% *w*/*v*) were prepared: maltodextrin (MD), free hydrolysate (H), and microencapsulated hydrolysate (HEn). For determining the viscoelastic moduli, time sweeps at 20 °C for 1800 s at 0.05 Hz and small stress (σ = 1.5 Pa) were performed to minimize structural changes.

Flows were characterized by the step test, with three intervals starting with a pre-shear interval to homogenize the suspensions (100 s^−1^ for 300 s). The three steps were: (1) reference interval (150 s^−1^ for 90 s); (2) high shear-rate interval (1000 s^−1^ for 45 s) to damage the internal structure; (3) regeneration interval (150 s^−1^, 600 s) at 20 °C. Each rheological test was repeated five times.

### 2.6. Microbiological Analysis

Yogurts were evaluated for microbiological analysis according to the method described by Arancibia et al. [29]. Aliquots of approximately 10.0 ± 0.2 g were weighed and transferred to sterile polyethylene bags (Sterilin, Stone, UK) with 90 mL of 0.1% (*w*/*v*) sterile peptone water (Oxoid, Unipath Ltd., Basingstoke, UK) and homogenized for 1 min medium speed in a Stomacher (Colworth 400, Seward, UK) at room temperature. Then, appropriate dilutions were prepared for the following bacteriological determinations: (i) *Streptococcus thermophilus*, on spread plates of ESTY agar + lactose (0.5%) (Pronadisa, Spain) and (ii) *Lactobacillus delbruckii* sp. *bulgaricus*, on spread plates of MRS Agar (Pronadisa, Spain) + tween 80 (0.1%) (Sigma-Aldrich, Darmstadt, Germany) + Cysteine (0.05%) (Sigma-Aldrich), both incubated at 42 °C for 24 h in an anaerobic jar (Oxoid), (iii) *Enterobacteriaceae*, on pour double-layered plates of Violet Red Bile Glucose Agar—VRBG (Oxoid), incubated at 30 °C for 48 h, and (iv) molds and yeasts on spread plates of potato dextrose agar (Scharlab, Spain) incubated at 25 °C for 72 h. These analyses were carried out on the day the yogurts were prepared to confirm that a suitable product was obtained. All determinations were conducted in duplicate.

### 2.7. Yogurt Bioactivity

For the analysis of antioxidant and antihypertensive activities, the supernatant of yogurt was used, which was prepared according to Zhang et al. [30]. For that, 10 g of yogurt samples were centrifuged at 4330× *g* for 5 min at 4 °C, and then the supernatants were recentrifuged under the same conditions.

#### 2.7.1. Antioxidant Activity

##### ABTS Radical Scavenging Activity

The 2,2′-azinobis-(3-ethylbenzothiazoline-6-sulfonic acid) (ABTS) radical scavenging activity was determined using the methods detailed by Zheng et al. [31], with modifications. The stock solution of the ABTS radical was generated by incubating 7 mM ABTS with 140 mM potassium persulfate in the dark for 16 h at room temperature. Before use, the stock solution was diluted with phosphate-buffered saline (PBS, pH 7.4) to an absorbance of 0.70 ± 0.02 at 734 nm. A 50 µL aliquot of the yogurt supernatant (diluted 1:5 in PBS) was homogenized with 150 µL of ABTS, and after reaction at 30 °C for 6 min in the dark, the absorbance was measured at 734 nm. The ABTS radical scavenging activity was calculated as follows: [(*A_c_ − A_s_*)/*A_c_*] × 100, where *A_c_* is the absorbance of the control, reagents without sample, and *A_s_* is absorbance with sample. The determination was carried out in triplicate.

##### Reducing Power

The reducing power was assessed as described by Canabady-Rochelle et al. [32], with modifications. An aliquot of 70 µL of yogurt supernatant (diluted 1:5 in phosphate buffer pH 6.6, 200 mM) was homogenized with 35 µL of potassium ferricyanide solution (1% *w*/*v*), and incubated at 50 °C for 20 min. Subsequently, 135 μL of distilled water, 33 μL of trichloroacetic acid (10% *w*/*v*) and 27 μL of ferric chloride (0.1% *w*/*v*) were added and after 10 min, the absorbance was measured at 700 nm. The reducing power of the sample was shown as the absorbance at 700 nm after subtracting the absorbance value from the blank, where a higher absorbance value indicates greater reducing power. The determination was carried out in triplicate.

#### 2.7.2. ACE Inhibitory Activity

The ability of the sample to inhibit the angiotensin-converting enzyme (ACE) was determined according to Alemán et al. [33], with some modifications. The reaction was composed of 50 µL of 5 mM Hipuryl-histidyl-leucine (HHL), 80 µL of ACE (0.025 U/mL) and 20 µL of the yogurt supernatant (diluted 1:5 in 100 mM potassium phosphate buffer, containing 300 mM NaCl, pH 8.3). The determination was performed by reverse phase High Performance Liquid Chromatography (RP-HPLC) (model SPE-MA10AVP, Shimadzu, Kyoto, Japan). The injection volume was 50 µL and the flow rate 0.8 mL/ min, using an acetonitrile gradient from 20% to 60% in 0.1% trifluoroacetic acid (TFA) (*v*/*v*) for 26 min. The results were expressed as % of ACE-inhibitory activity.

### 2.8. Sensory Analysis

The sensory analysis of the formulated yogurts was conducted at the Instituto de Ciencia y Tecnología de Alimentos y Nutrición (ICTAN-CSIC, Madrid, Spain) with 10 semi-trained judges of both sexes, selected from among students and researchers of the Institute. The specific attributes that the panel was asked to classify in the yogurts were the following: color, flavor, odor, texture and acidity. The scale values ranged from 0 to 9. Number 9 was assigned to the most positive terms, while number 0 was assigned to the least desirable attributes/characteristics. Regarding texture and acidity, number 9 was assigned to firm and very acid, respectively. Sensory tests were performed in individual booths with lighting control; samples were served in disposable plastic cups, encoded with 3-digit numbers obtained from a table of random numbers. Mineral water was also provided for cleansing the palate between the evaluations of the different samples of yogurt.

### 2.9. Statistical Analysis

The data were submitted to analysis of variance (ANOVA) and the means compared by the *t*-Student test (to compare between day 1 and day 7) or the Tukey test with a significance level of 5% using Statistica software (StatSoft, Inc., Tulsa, OK, USA).

## 3. Results and Discussion

### 3.1. Visual Appearance, Proximal Analyses and Physicochemical Properties of Yogurts

**Figure 1:**
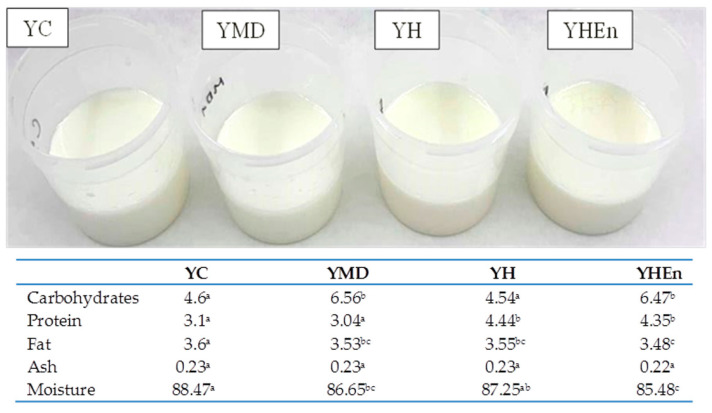
Visual appearance and proximate composition of the different yogurts. YC: control yogurt; YMD: yogurt with addition of maltodextrin; YH: yogurt with addition of free protein hydrolysate; YHEn: yogurt with addition of microencapsulated protein hydrolysate. Different letters (a,b,c) indicate significant differences between samples within the same line (*p* < 0.05).

Figure 1 shows the visual appearance of the different yogurts. To the naked eye, all yogurts presented a similar color, with similar gel-like appearance and no evidence of whey separation. The proximate composition revealed that the yogurts were similar, with significant differences (*p* < 0.05) mainly in carbohydrate and protein content due to the incorporation of maltodextrin and hydrolysate in some yogurts.

The pH, titratable acidity (TA), color and syneresis of the yogurts after 1 and 7 days of storage under refrigeration, are shown in Table 1. The lowest pH value after 1 and 7 days of storage was observed in the yogurt with the addition of the microencapsulated hydrolysate (YHEn) while the highest pH observed was in the yogurt with the addition of MD (YMD), (*p* < 0.05). The pH values of MD, the free hydrolysate and microencapsulated hydrolysate were 5.7, 7.03 and 6.91, respectively. Hence, it seems that the pH of the yogurts was probably not affected by the addition of these ingredients. Moreover, a decrease in pH was observed in all yogurts throughout storage, which can be mostly attributed to the production of microbial metabolites. Similarly, a decrease in pH was also observed by Abdel-Hamid et al. [9] when supplementing probiotic yogurt with 1% and 2% monk fruit extract (*Siraitia grosvenorii*). These authors reported that supplementation with the extract may have stimulated bacterial growth. Notably, fermented products with pH in the range of 4.2 and 4.4 are preferred by consumers [34]. In the present work, these pH ranges were achieved in yogurts after 7 days of storage (Table 1).

The titratable acidity of the yogurts varied from 0.73% to 1.09% (Table 1), the YH and YHEn yogurts showing the highest values, both after 1 and 7 days of storage. In addition, with the exception of YC, there was an increase in the titratable acidity with storage time (*p* < 0.05). Similar results were reported by Córdova-Ramos et al. [35] when studying the addition of jumbo squid powder (*Dosidicus gigas*) using maltodextrin as an encapsulating agent by spray drying (MD with different DE (11) and drying conditions) in yogurt. The authors verified an increase in acidity as squid powder concentration increased (1%, 3%, 5%, 7% and 10%), ranging from 0.76% to 1.05% in the yogurt without squid powder and with the highest concentration, respectively. An increase in titratable acidity was also observed in yogurts supplemented with pineapple peel powder or inulin after 28 days of storage [36]. According to the authors, the supplementation increased the acidifying capacity of the starter cultures during storage.

Color is the first characteristic perceived by consumers, and it can influence their preference, so it is an important attribute to be evaluated [37]. The color parameters (L*, a* and b*) of the different formulated yogurts are shown in Table 1. A decrease in lightness values (L*) was observed for yogurts with the addition of the different tested ingredients, and therefore, the highest values observed for YC also reflected a higher whiteness index. As also observed by Silva et al. [5] such a phenomenon may have occurred because this batch did not have any powder ingredients. Conversely, on the same evaluation day, the YH and YHEn yogurts tended to show higher values for yellowness (b*) (*p* < 0.05), probably due to the color of the hydrolysate. Carmona et al. [38] verified that encapsulating yellow–orange cactus pear Opuntia ficus-indica pulp, using maltodextrin (MD) as an encapsulating agent, protected the quality of the pigment (thus allowing its use as a yellow colorant for yogurt) and that, additionally, maltodextrin did not negatively affect b* and a* parameters in the yogurt when compared with other treatments.

The YMD sample showed differences (*p* < 0.05) in a* values compared with YH after 1 day of storage and with YH and YHEn after 7 days of storage, but was similar to YC (*p* > 0.05). Regarding storage, in general the L* parameter showed a slight increase over time, while a* and b* values did not change, except in YHEn, which did not present any change in relation to the L* parameter and showed a slight decrease in a* values. The whiteness index (WI) was in the same range in all samples, decreasing less than 2% compared with the control (*p* < 0.05).

Syneresis is an important parameter in yogurt since it can affect its quality during storage through the accumulation of serum on the surface, influencing the acceptability of the product [4,28,30]. The addition of the different ingredients provided a decrease in syneresis, with the lowest values observed in YHEn, independently of storage time (*p* < 0.05) (Table 1). In general, syneresis was not influenced by storage time, with the exception of YH, which showed a small decrease after day 7 (*p* < 0.05). Córdova-Ramos et al. [35] reported a decrease in syneresis in yogurts with the addition of different concentrations (1, 3, 5, 7 and 10 g/100 mL) of jumbo squid powder (*Dosidicus gigas*) obtained by spray drying using MD as encapsulating agent. The decrease in syneresis was associated with the functional properties of the protein, since protein and MD facilitate water retention, thus preventing this phenomenon. These authors reported that the lowest syneresis values were observed in yogurts with 7 and 10 g/100 mL of the powder (4.20% and 1.0%, respectively) while the addition of 3 g/100 mL presented a syneresis percentage (9.10%) similar to that obtained in the present study in YHEn (Table 1). In addition, the lowest syneresis was found in YHEn. This result was similar to that reported by Demirci et al. [6] in yogurts with the addition of different concentrations of rice bran (1, 2 and 3%), which ranged from 9.79% to 8.80%, while the control yogurt presented 10.29%. The authors related the decrease in syneresis to the water-retention capacity of rice bran dietary fibers. In the present work, the reduction in the syneresis of formulated yogurts may be associated with the water-retention capacity of the protein hydrolysate by ion-dipole interactions. This fact may be due to the amino acid profile, which has a predominance of hydrophilic amino acids (57.4%) and negatively charged amino acids (64.4%). Moreover, the reduction in syneresis was enhanced in the yogurt to which the microencapsulated hydrolysate was added, possibly because MD also contributed to increase water retention by hydrogen bonding and dipole–dipole interactions.

### 3.2. Textural and Viscoelastic Properties

Texture is an important attribute regarding the quality of yogurt [37]. The firmness and cohesiveness of the different yogurts after 1 and 7 days of storage are shown in Table 2. The addition of the hydrolysate to the yogurts, both free and microencapsulated, resulted in a slight decrease in firmness compared to the YC sample (*p* < 0.05), representing approximately 11% (YH) and 10% (YHEn) after 1 day, and 30% (YH) and 19% (YHEn) after 7 days of storage. On the other hand, YH and YHEn showed increases in cohesiveness (*p* < 0.05) of approximately 12% and 15%, respectively, after 1 day and ~92% and 18%, respectively, after 7 days of storage compared with YC. After 7 days YH showed the highest cohesiveness values (*p* < 0.05). In general, the firmness and cohesiveness of the different yogurts were not significantly influenced by storage time, except for YC and YH, which showed greater firmness and greater cohesiveness, respectively, after 7 days of storage.

Barkallah et al. [4] studied the addition of different concentrations of *Spirulina platensis* (0.25%, 0.5%, 0.75% and 1.0%) to yogurt and observed greater firmness in the control yogurt and with the addition of 0.25% of *Spirulina platensis* (0.67 and 0.62 N, respectively). The addition of higher concentrations resulted in yogurts with lower firmness values which, according to the authors, was due to the interruption of gel formation as concentrations of microalga were increased. In the same study, the authors found no differences in relation to cohesiveness for the samples. It is worth noting that both firmness and cohesiveness values were lower than those observed in the present study.

Additionally, Öztürk et al. [39], when studying the fortification of set-type yogurts with peeled or unpeeled oleaster (*Elaeagnus angustifolia* L.) flours (1% and 2%), reported a reduction in firmness and an increase in cohesiveness on the first day of storage compared to the control yogurt. According to the authors, the control yogurt showed a longer fermentation time resulting in greater firmness, while the increase in cohesiveness would occur due to the water retention capacity of the flours added into the protein matrix. During fermentation, the formation of lactic acid by the action of microorganisms occurs, and the pH drop induces the aggregation of casein and the formation of disulfide bonds between denatured whey proteins and k–casein, resulting in the characteristic gel formation, texture and properties of yogurt [37,40,41].

In the present study, the yogurts with the addition of the free and microencapsulated hydrolysate required less time to ferment (about 60–90 min) compared with the control yogurt or that with MD; this factor may have influenced the lower firmness values found. Moreover, the addition of these ingredients may have caused an interruption of gel formation, reducing firmness. Parallel to this, the greater cohesiveness may be associated with the water retention capacity, mainly due to the functional properties of protein hydrolysates [42].

**Figure 2:**
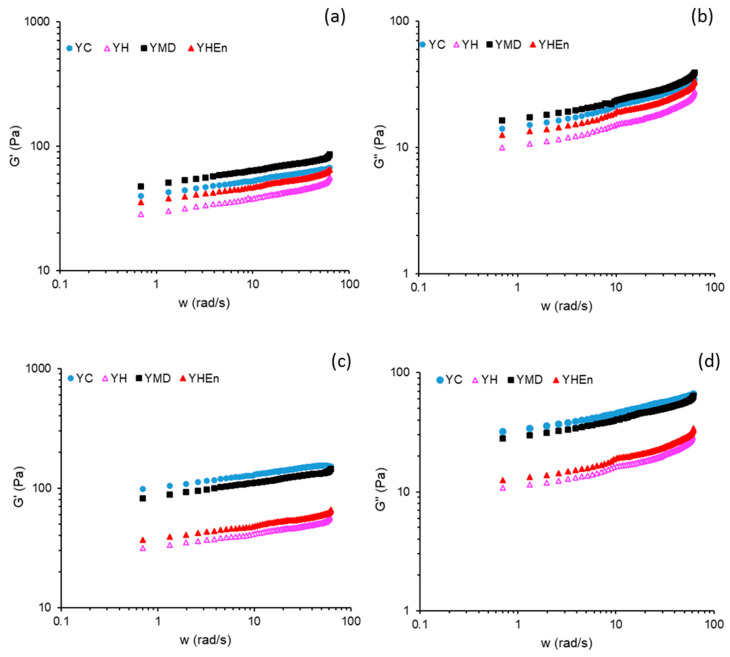
Mechanical spectra of yogurts during storage at 5 °C. Storage modulus −*G*′ at 1 day (**a**); loss modulus −*G*″ at 1 day (**b**); storage modulus −*G*′ at 7 days (**c**); loss modulus −*G*″ at 7 days (**d**). YC: control yogurt; YMD: yogurt with addition of maltodextrin; YH: yogurt with addition of free protein hydrolysate; YHEn: yogurt with addition of microencapsulated protein hydrolysate.

The viscoelastic properties of the yogurt samples were evaluated by small-amplitude oscillatory shear (SAOS) tests. Mechanical spectra of the different yogurts after 1 and 7 days of storage showed typical gel-like behaviour, with *G*′ > *G*″ along the whole frequency range (Figure 2). The power law model may be used to fit both *G*′ and *G*″ with the angular frequency ω (Equations (2) and (3)):

(2) $$G^{\prime }=G_{0}^{\prime }\cdot \omega^{n^{\prime }}$$

(3) $$G^{\prime \prime }=G_{0}^{\prime \prime }\cdot \omega^{n^{\prime \prime }}$$

where *G*_0_′ and *G*_0_″ are the respective storage and loss moduli at 1 rad/s, and *n*′ and *n*″ exponents denote the viscoelastic response in terms of the time stability of both *G*′ and *G*″ at short time scales. The viscoelastic parameters resulting from Equations (2) and (3) (*G*_0_′ and *G*_0_″) are both a measurement of the gel strength of samples since they provide the complete (elastic and viscous) deformation resistance [43].

After day 1, the yogurts with the free hydrolysate (YH) and microencapsulated hydrolysate (YHEn) exhibited the lowest values for *G*_0_′ and *G*_0_″ (Table 2). This result indicates that the hydrolysate, in both forms, reduced the gel strength of the casein matrix, maintaining a similar degree of viscoelasticity as was evidenced by the similar values for the loss factor at 1 rad/s (*tanδ*) (Table 2). This result could be explained by the fact that the hydrolysate introduces a negative electric charge, consequently increasing the electrostatic repulsive forces within the micellar structure, which expand the network, enhancing the hydration of the gel network [44]. This result is consistent with the observed decrease in firmness in YH and YHEn as compared with YC from textural analysis.

The yogurt with maltodextrin (YMD) exhibited slightly higher values for *G*_0_′ and *G*_0_″ than those in YC after day 1. The electrostatic neutral character of these glucose polymers would favor the mutual associations between maltodextrin and casein by hydrogen bonding and dipole–dipole interactions, showing a certain binder role for MD in the yogurt matrix. This result is consistent with the lower *tanδ* value for YMD vs. YC (Table 2), which suggests greater strength in the intermolecular interactions and consequently a longer bond lifetime in the gel matrix [45].

As regards *n*′ and *n*″ exponents, it might be observed that for all samples, *n*″ > *n*′, this means that the rate of decrease of *G*″ with decreasing ω, was higher than that for *G*′, resulting in a shear-induced gelation at lower frequencies (higher oscillation times) [46]. This fact shows a shear-induced increase in the energy stabilization of network bonds at lower frequencies, compatible with the decrease in the gel strength at higher oscillation times [47].

After 7 days of storage, *G*_0_′ and *G*_0_″ values increased in YC and YMD compared with those found after day 1. This result was consistent with the observed increase in firmness from textural analysis, indicating considerable gel reinforcement during storage, which would be explained by the natural strengthening of the dipolar interactions and hydrogen bonds in the casein matrix induced by cool storage [44]. This effect was partially mitigated by the presence of maltodextrin, which would stabilize the structural rearrangements in the casein matrix during storage. In addition, both *G*_0_′ and *G*_0_″ were scarcely modified in YH and YHEn during the storage period, showing the stabilizing role of the hydrolysate in the casein matrix, especially in microencapsulated form.

The increase in both *G*_0_′ and *G*_0_″ after 7 days was consistent with the observed increase in L* (Table 1). L* shows the light scattered by various structural elements (casein aggregates, molecular fragments, etc.). After 7 days, the gel strength of the different networks increased moderately, so that denser matrices were formed, enhancing the diffuse reflection of electromagnetic waves and consequently increasing L* [45].

In general, *n*′ and *n*″ values decreased after day 7 of storage, maintaining a similar positive difference between *n*″ and *n*′ (Table 2). Therefore, the lower exponents (*n*′ and *n*″) indicate an improvement in stability in the four yogurt networks over time, maintaining a similar stabilization energy at lower frequencies than on day 1.

**Figure 3:**
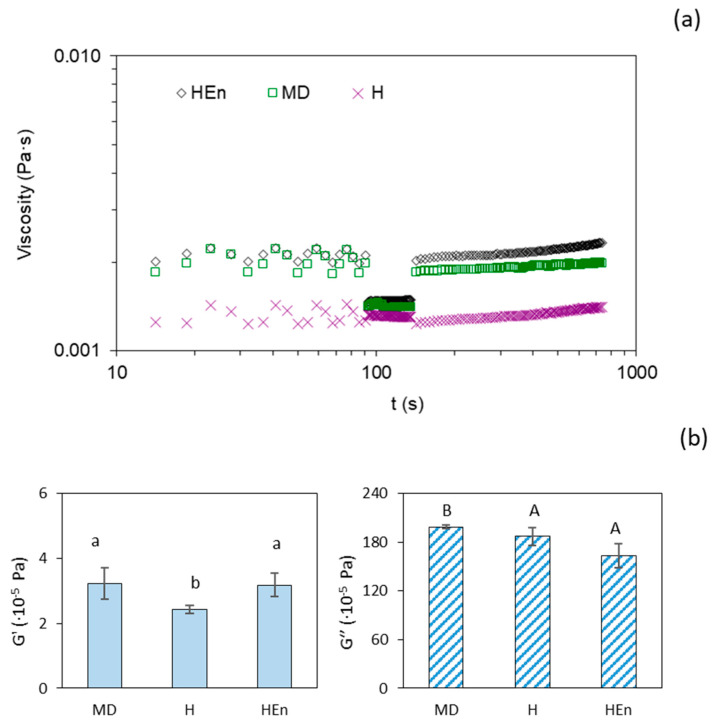
Evolution of viscosity values for the three-step flow (**a**) and viscoelastic parameters (**b**) for aqueous solutions at 1.5% concentration of maltodextrin (MD), hydrolysate (H) and microencapsulated hydrolysate (HEn) at 20 °C. Different letters (a,b) or (A,B) indicate a significant difference between samples (*p* < 0.05).

In order to gain insight into the rheological differences among the three ingredients (H, MD and HEn) in model systems, i.e., outside the protein gel matrix environment at the same concentration, the flow behaviour and the viscoelastic characteristics of diluted aqueous solutions (1.5%, *w*/*v*) were also analyzed (Figure 3). Such a low concentration was selected to resemble the low concentration of the hydrolysate in the yogurt matrix. The dissolved maltodextrin (MD) and the microencapsulated hydrolysate (HEn) presented a similar trend (shear thinning flow) and similar viscosity values during the three-step flow, while the opposite was observed in the free hydrolysate, which exhibited the lowest viscosity and virtually Newtonian behaviour, as was evidenced by the stationary values of viscosity in the three steps (Figure 3a). The dynamic oscillatory test showed an evident fluid-like response in the three samples based on noticeably higher values for *G*″ compared with those for *G*′ (Figure 3b). Both MD and HEn solutions showed considerably higher *G*′ values compared with the H aqueous solution, while showing no significant differences between each other. This result was consistent with the decrease in viscosity in the second step, and their regeneration ability in the third step, attributed to the contribution of maltodextrin (Figure 3a). In contrast, *G*″ decreased in H, and more evidently in HEn, with respect to plain MD (Figure 3b). These findings reflect a lower level of intermolecular association at small oscillatory shear in HEn, and suggest that microcapsules may be less prone to interacting with each other and also with the surrounding medium, explaining why this preparation in yogurt resulted in more stable samples, which behaved differently compared with plain MD yogurts.

### 3.3. Microbiological Analysis

To confirm that the process of obtaining the yogurts was properly carried out, specific counts of the two major bacterial species present in yogurt were determined. The counts of *S. thermophilus* were YC: 8.72 ± 0.10 log CFU/g; YMD: 8.71 ± 0.10 log CFU/g; YH: 8.82 ± 0.04 log CFU/g; YHEn: 9.20 ± 0.01 log CFU/g (*p* < 0.05). Thus, the addition of the microencapsulated hydrolysate seems to have favored the growth of *S. thermophilus*, which could explain the lower pH and higher acidity compared with the YC sample. Regarding *L. bulgaricus*, counts were slightly higher in YH (YC: 6.42 ± 0.11; YMD: 6.92 ± 0.06; YH: 7.25 ± 0.09; YHEn: 6.94 ± 0.09 log UFC/g) (*p* < 0.05), indicating that the incorporation of the hydrolysate favored the growth of the starter culture in some way. YHEn registered higher values than the control yogurt, and similar values to those of YMD (*p* < 0.05), which indicated that maltodextrin, as a glucose supply, could also promote in some way the growth of this group of microorganisms. The viable counts of *S. thermophilus* were significantly more numerous than those of *L. bulgaricus* in all samples (*p* < 0.05). Zhao et al. [48] verified the same tendency in yogurts elaborated with α-lactalbumin hydrolysate-calcium (α-LAH-Ca) complexes.

The relatively low pH of yogurt and other fermented dairy products is a result of the fermentation of milk lactose into lactic acid, caused by the activity of lactic acid bacteria usually added as starter cultures [49,50]. In this study, as shown in Table 1, the pH of the yogurts evaluated ranged from 4.35 to 4.61. Furthermore, the yogurt starter cultures contained *Streptococcus thermophilus* and *Lactobacillus delbrueckii* ssp. *bulgaricus*, which largely out-compete other bacteria present in milk due the inhibitory effect of lactic acid and the utilization of the primary carbohydrate source (lactose), as well as the production of other inhibitory compounds [49,50].

In this study, the presence of *Enterobacteriaceae*, molds and yeasts was not found after 1 and 7 days of storage (data not shown). There is growing evidence that the *Enterobacteriaceae* family can accurately reflect the hygienic conditions of processing milk and its derivatives, even during storage, despite the widespread use of coliforms as an indicator of hygienic–sanitary conditions. In addition, the presence of molds and yeasts in yogurt is indicative of unsatisfactory sanitary practices in manufacturing or packaging [49]. Accordingly, Barkallah et al. [4] did not observe the presence of the mentioned microorganisms in yogurt with the addition of *Spirulina platensis*.

### 3.4. Bioactivity

#### 3.4.1. Antioxidant Activity

Antioxidant compounds can contribute to health promotion by eliminating free radicals, such as reactive oxygen species, as well as to increase the shelf life of foods, slowing down the process of lipid oxidation [36]. Yogurt per se, in general, may present antioxidant activity in view of its content of bioactive peptides, as reported in previous studies [6,9,36,39].

**Figure 4:**
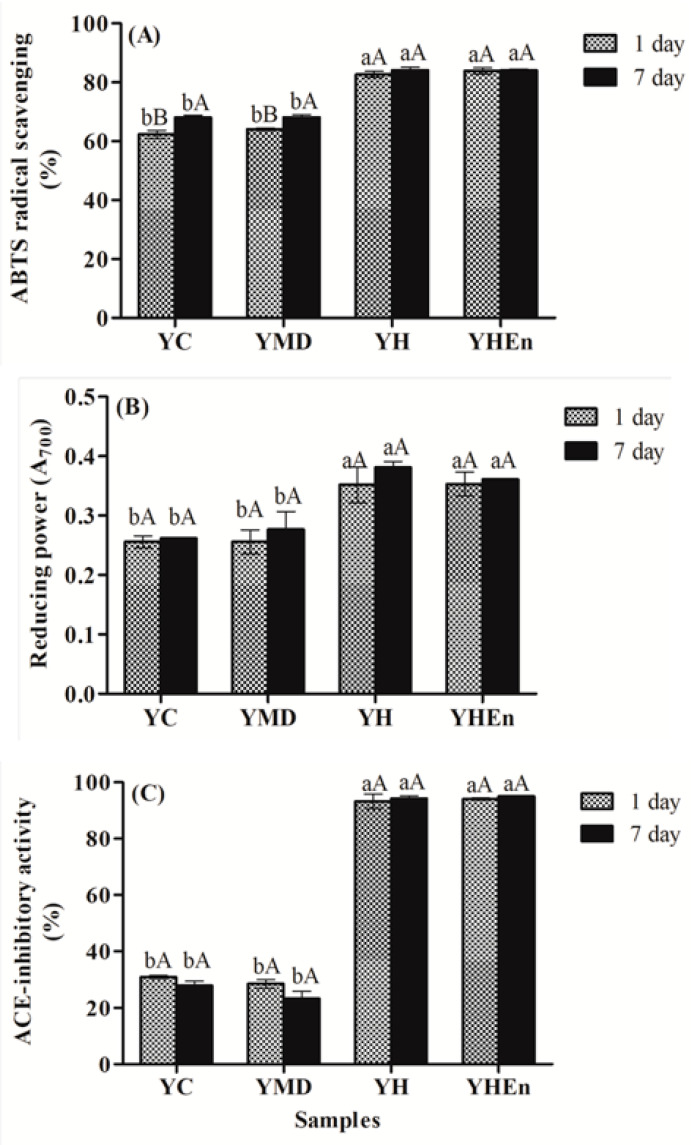
ABTS radical scavenging activity (**A**), reducing power (**B**) and ACE inhibitory activity (**C**) of yogurts during storage. Different lowercase letters (a,b) indicate a significant difference between samples for the same day (*p* < 0.05). Different uppercase letters (A,B) indicate a significant difference for the same sample on different days (*p* < 0.05).

The antioxidant activity of the different yogurts after 1 and 7 days of storage was evaluated by assessing the ABTS radical scavenging activity and reducing power (Figure 4A and Figure 4B, respectively). The addition of the free and microencapsulated hydrolysate to yogurts (YH and YHEn, respectively) increased the ABTS radicals scavenging activity (*p* < 0.05) in comparison with the YC and YMD samples, which showed a similar behavior (*p* > 0.05). The increase in antioxidant activity was probably due to peptides with antioxidant activity present in the protein hydrolysate of stripped weakfish, as previously reported by Lima et al. [25]. The activity of the hydrolysates was maintained, both in the free and microencapsulated forms in the yogurt matrix. These findings are in line with those reported by Abdel-Hamid et al. [9] and Demirci et al. [6], who found an increase in the antioxidant activity of yogurts with the addition of the fruit extracts *Siraitia grosvenorii* and rice bran, respectively. According to the authors, this was due to the composition of added phytochemicals with antioxidant activity. Additionally, Silva et al. [5] evaluated the antioxidant activity of yogurts functionalized with *Spirulina platensis* in free form and microencapsulated with MD, or with MD cross-linked with citric acid obtained by spray drying, reporting greater antioxidant activity (DPPH assay) for both functionalized yogurts compared with the control yogurt (especially in the case of the sample with MD cross-linked with citric acid). According to the authors, the addition of *Spirulina platensis* in the encapsulated form presents advantages such as masking the unpleasant fishy odor. Similarly, in the mentioned study, no differences in antioxidant activity were observed in terms of storage time (4 and 7 days).

Francisco et al. [7] reported a decrease in antioxidant activity during storage (0–7 days) for yogurts with added mushroom extracts (*Agaricus bisporus*) in free form and encapsulated by spray drying with MD cross-linked with citric acid (thermally untreated forms), while the opposite behavior was observed in thermally treated microencapsulated extracts after atomization. This decrease throughout storage time may be associated with the degradation of the extract when incorporated in free form by direct contact with the food matrix, and when thermally untreated, by the rapid release of the microencapsulated extract to the food matrix with the subsequent degradation.

In the present study there was no degradation of bioactive peptides, possibly because the antioxidant activity (ABTS and reducing power) remained stable throughout storage (*p* > 0.05). It should be noted that YH and YHEn did not differ significantly in either evaluated property (*p* > 0.05) (Figure 3). Thus, the incorporation of the microencapsulated hydrolysate may be an alternative for increasing the antioxidant activity of yogurts while possibly masking any potential changes to odor and flavor.

#### 3.4.2. ACE Inhibitory Activity

Angiotensin-I converting enzyme (ACE) plays an important role in regulating blood pressure, as it produces the potent vasoconstrictor angiotensin II and degrades a vasodilator called bradykinin, causing an increase in blood pressure [51,52].

Fermented dairy products can provide beneficial health effects by releasing peptides with ACE inhibitory activity as a result of the proteolysis of milk proteins during fermentation [8,53]. Figure 4C shows the ACE inhibitory activity after 1 and 7 days of storage. According to these results, the activity presented by YH and YHEn was more than three-fold greater compared with that of YC or YMD (*p* < 0.05). In addition, the effect of the storage time can be considered negligible in these yogurts since the ACE inhibitory activity remained stable throughout the 7 days of storage (*p* > 0.05).

The results obtained in the present study are consistent with those observed by Abdel-Hamid et al. [9] who reported a significant increase in the ACE inhibitory activity of probiotic yogurts supplemented with *Siraitia grosvenorii* fruit extract (73.36–81.39% compared to the 62.05% control). The mentioned authors correlated this activity with the degree of hydrolysis (proteolysis) of the supplemented yogurt, in which the release of small peptides occurs. In another study, Wulandani et al. [10] evaluated the ACE inhibitory activity of yogurts added with *Ficus glomerata* Roxb fruit extract (5% and 10%) during cold storage for 28 days, reporting the greatest activity on the seventh day of storage in yogurt with the addition of 10% *F. glomerata Roxb* extract (69.11 ± 0.50%) compared with yogurt without *F. glomerata Roxb* extract (53.47 ± 1.07%). Shori et al. [8] also found an increase in the ACE inhibitory activity in freshly made yogurts to which fish collagen was added compared with the control yogurt, presenting approximately 53% and 40% more inhibition, respectively. It is worth mentioning that the reported values in yogurts with the addition of fruits were lower than those observed in the present study.

Thus, the results in the present study suggest that the hydrolysate, both free and microencapsulated, is a good source of ACE inhibitor peptides, providing yogurt with increased ACE inhibitory activity after at least 7 days of storage.

### 3.5. Sensory Analysis

**Figure 5:**
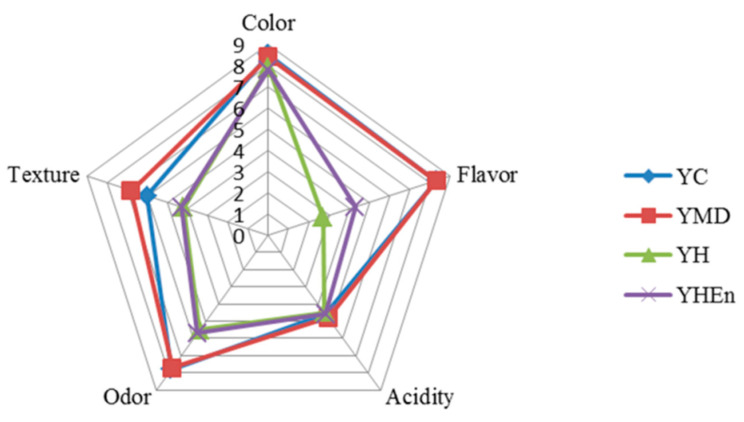
Sensory evaluation of different yogurts.

The addition of new ingredients to yogurt can cause possible interferences, mainly in regard to taste and texture. Figure 5 shows the sensory profile of the different yogurts as evaluated by the panelists. According to the results, there were no significant differences among yogurts for color and acidity (*p* > 0.05). Moreover, the presence of MD did not affect the organoleptic properties of yogurt in any of the evaluated attributes.

The addition of the free and microencapsulated protein hydrolysate (YH and YHEn) affected the texture, odor and flavor compared with the control yogurt (*p* < 0.05). Although they presented a significant difference in relation to YC, YH and YHEn showed an intermediate score in relation to texture; the panelists described the texture of YH and YHEn as slightly softer than that of the control but without losing the characteristic gel-like consistency of yogurt.

Regarding odor, a slightly fishy or peculiar smell was detected, especially in YH. Among the sensory attributes evaluated, the lowest scores obtained corresponded to flavor. However, YHEn showed a significant difference compared with YH (*p* < 0.05), presenting a higher score, and thus suggesting that encapsulation masked the fishy flavor. In the observations, the panelists indicated that the yogurts with the free and microencapsulated hydrolysates had a different flavor compared with that of a traditional yogurt (control), but were also different from each other. A more intense fishy flavor was identified in the yogurt with the free hydrolysate (YH), while YHEn was perceived as having a different flavor from that of the control, which was, however, undefined.

Taking into account the sensory attributes of a traditional yogurt, a very familiar product to consumers, it is frequently and well documented that the incorporation of ingredients (other than fruits) modifies or is detrimental to the organoleptic characteristics. Accordingly, the addition of *Allium sativum* to yogurt lowered the score for wateriness, aroma and taste (in terms of sourness) compared with the control yogurt [8]. Demirci et al. [6] reported that the addition of 1%, 2% and 3% rice bran negatively affected the appearance, texture, taste and odor of the yogurt. In the formulation of yogurt with different concentrations of jumbo squid powder (1%, 3%, 5%, 7% and 10%), significant differences in relation to color, taste, and texture were reported, while the addition of 3% did not differ statistically from the controls [35]. Barkallah et al. [4] stated that there was no significant difference in flavor between the control yogurt and that containing 0.25% *Spirulina platensis*, however, the addition of 1% had the lowest score for flavor.

In the present study, although there were differences in texture and flavor, the addition of microencapsulated hydrolysate was positive, as it masked the fish flavor while maintaining its bioactivity. There are many types of yogurt on the market; the consumer chooses among the available options, including fortified yogurt with bioactive products, which constitutes a new product. Thus, while the judges were able to differentiate the yogurts containing hydrolysates, this did not imply that the quality was worse, but rather that the yogurts were different with respect to the control; therefore, the acceptability parameter was excluded in the evaluation so as to prevent a biased judgement based on familiarity. Nevertheless, more studies should be conducted in order to improve these sensorial attributes in supplemented yogurt with fish protein hydrolysates.

## 4. Conclusions

Quality yogurts were made with the incorporation of a protein hydrolysate, free or microencapsulated with maltodextrin. These ingredients led to a reduction in syneresis, especially for the yogurt with the added microencapsulated hydrolysate. Yogurts with hydrolysates showed a slight reduction in firmness, while they were slightly more cohesive compared with the control yogurt. Both free and microencapsulated hydrolysates caused a reduction in the viscoelastic parameters, maintaining the characteristic gel-like structure of yogurt, and provided greater rheological stability after one week of storage, especially in the microencapsulated form. In addition, the incorporation of hydrolysates in both forms resulted in yogurts with greater antioxidant and antihypertensive activities, which were maintained after 7 days of storage. The antioxidant and antihypertensive activities shown in vitro by a functional food, such as yoghurt made with probiotic bacteria and further enhanced by the incorporation of protein hydrolysates, provide such a product with a potential health effect, although in vivo studies are needed to demonstrate this effect (yogurt behavior once digested, bioavailability studies, etc.). If the health effect is proven, the inclusion of yogurt in a diet could reduce the need for medication to regulate, for example, blood pressure. Although there were differences in texture, odor and flavor, the incorporation of the hydrolysate in microencapsulated form showed advantages over the free form in relation to the masking of the fish flavor, without altering the bioactive properties, and thus a yoghurt containing this addition may constitute an acceptable fortified food.

## Figures and Tables

**Table 1:** Physicochemical properties of yogurts during storage.

Parameters	Time (Days)	Samples			
		YC	YMD	YH	YHEn
pH	1	4.58 ± 0.01 ^bA^	4.61 ± 0.01 ^aA^	4.57 ± 0.01 ^bA^	4.53 ± 0.02 ^cA^
	7	4.38 ± 0.01 ^bB^	4.43 ± 0.01 ^aB^	4.37 ± 0.01 ^bB^	4.35 ± 0.01 ^cB^
TA (% lactic acid)	1	0.73 ± 0.01 ^bA^	0.73 ± 0.01 ^bB^	0.94 ± 0.02 ^aB^	0.95 ± 0.01 ^aB^
	7	0.77 ± 0.04 ^bA^	0.75 ± 0.01 ^bA^	1.08 ± 0.01 ^aA^	1.09 ± 0.02 ^aA^
L*	1	83.25 ± 0.12 ^aB^	82.64 ± 0.10 ^bB^	82.37 ± 0.07 ^bcB^	82.09 ± 0.19 ^cA^
	7	83.59 ± 0.04 ^aA^	83.30 ± 0.15 ^bA^	82.80 ± 0.11 ^cA^	82.45 ± 0.03 ^dA^
a*	1	−1.53 ± 0.06 ^abA^	−1.57 ± 0.03 ^bA^	−1.42 ± 0.03 ^aA^	−1.47 ± 0.04 ^abA^
	7	−1.49 ± 0.02 ^bcA^	−1.54 ± 0.03 ^cA^	−1.43 ± 0.03 ^abA^	−1.42 ± 0.02 ^aB^
b*	1	6.11 ± 0.08 ^bA^	6.21 ± 0.10 ^bA^	7.16 ± 0.04 ^aA^	7.22 ± 0.08 ^aA^
	7	6.20 ± 0.06 ^bA^	6.20 ± 0.07 ^bA^	7.26 ± 0.10 ^aA^	7.26 ± 0.05 ^aA^
WI	1	82.10 ± 0.12 ^aB^	81.50 ± 0.11 ^bA^	80.92 ± 0.06 ^cB^	80.63 ± 0.21 ^cA^
	7	82.39 ± 0.06 ^aA^	82.12 ± 0.16 ^aA^	81.28 ± 0.14 ^bA^	80.96 ± 0.05 ^cA^
Syneresis (%)	1	13.97 ± 0.63 ^aA^	11.0 ± 0.45 ^bA^	11.10 ± 0.43 ^bA^	9.25 ± 0.19 ^cA^
	7	13.25 ± 1.12 ^aA^	9.89 ± 1.12 ^bcA^	10.53 ± 0.47 ^bB^	7.98 ± 0.79 ^cA^

TA: titratable acidity; L*: lightness; a*: red-green axis; b*: blue-yellow axis; WI: whiteness index; YC: control yogurt; YMD: yogurt with addition of maltodextrin; YH: yogurt with addition of free protein hydrolysate; YHEn: yogurt with addition of microencapsulated protein hydrolysate. Different lowercase letters (a,b,c,d) indicate a significant difference between samples for the same day (*p* < 0.05). Different uppercase letters (A,B) indicate a significant difference for the same sample on different days (*p* < 0.05).

**Table 2:** Textural and viscoelastic parameters (Equations (2) and (3)) of yogurts during refrigerated storage.

Parameters	Time	Samples			
	(Days)	YC	YMD	YH	YHEn
Firmness (N)	1	1.11 ± 0.02 ^aB^	1.13 ± 0.02 ^aA^	0.99 ± 0.03 ^bA^	1.00 ± 0.02 ^bA^
	7	1.34 ± 0.09 ^aA^	1.21 ± 0.10 ^abA^	0.94 ± 0.03 ^cA^	1.09 ± 0.08 ^bcA^
Cohesiveness	1	0.69 ± 0.02 ^bA^	0.71 ± 0.01 ^bA^	0.77 ± 0.00 ^aB^	0.79 ± 0.03 ^aA^
	7	0.67 ± 0.02 ^bA^	0.69 ± 0.04 ^bA^	1.29 ± 0.04 ^aA^	0.79 ± 0.11 ^bA^
*G*_0_′ (Pa)	1	40.71 ± 0.02	48.24 ± 0.06	27.62 ± 0.08	35.08 ± 0.07
	7	101.94 ± 0.09	85.00 ± 0.06	31.14 ± 0.11	36.98 ± 0.07
n′	1	0.116 ± 0.001	0.122 ± 0.001	0.144 ± 0.003	0.131 ± 0.002
	7	0.105 ± 0.001	0.117 ± 0.004	0.129 ± 0.004	0.121 ± 0.002
R^2^ (Equation (2))	1	0.998	0.988	0.958	0.972
	7	0.986	0.993	0.922	0.975
*G*_0_″ (Pa)	1	13.11 ± 0.06	14.71 ± 0.09	8.81 ± 0.08	11.24 ± 0.08
	7	30.72 ± 0.06	23.33 ± 0.08	9.39 ± 0.10	11.05 ± 0.09
n″	1	0.214 ± 0.003	0.208 ± 0.005	0.236 ± 0.006	0.226 ± 0.005
	7	0.177 ± 0.002	0.191 ± 0.003	0.239 ± 0.008	0.232 ± 0.006
R^2^ (Equation (3))	1	0.977	0.947	0.937	0.946
	7	0.988	0.976	0.913	0.946
tanδ	1	0.355 ± 0.007 ^aA^	0.343 ± 0.008 ^aA^	0.356 ± 0.007 ^aA^	0.358 ± 0.011 ^aA^
	7	0.333 ± 0.012 ^aB^	0.338 ± 0.005 ^aA^	0.343 ± 0.004 ^aB^	0.345 ± 0.011 ^aA^

YC: control yogurt; YMD: yogurt with addition of maltodextrin; YH: yogurt with addition of free protein hydrolysate; YHEn: yogurt with addition of microencapsulated protein hydrolysate. Different lowercase letters (a,b,c) indicate a significant difference between samples for the same day (*p* < 0.05). Different uppercase letters (A,B) indicate a significant difference for the same sample on different days (*p* < 0.05).

## Data Availability

All data relevant to the study are included in the article.
